# Potential of Piezoelectric MEMS Resonators for Grape Must Fermentation Monitoring †

**DOI:** 10.3390/mi8070200

**Published:** 2017-06-26

**Authors:** Georg Pfusterschmied, Javier Toledo, Martin Kucera, Wolfgang Steindl, Stefan Zemann, Víctor Ruiz-Díez, Michael Schneider, Achim Bittner, Jose Luis Sanchez-Rojas, Ulrich Schmid

**Affiliations:** 1 Institute of Sensor and Actuator Systems, TU Wien, 1040 Vienna, Austria; martin.kucera@tuwien.ac.at (M.K.); steindl.wolfgang@gmail.com (W.S.); stefan.zemann@gmx.at (S.Z.); michael.schneider@tuwien.ac.at (M.S.); achim.bittner@tuwien.ac.at (A.B.); ulrich.e366.schmid@tuwien.ac.at (U.S.); 2 Group of Microsystems, Actuators and Sensors, E.T.S.I. Industriales, Universidad de Castilla-La Mancha, 13071 Ciudad Real, Spain; javier.toledo.serrano@gmail.com (J.T.); victor.ruiz@uclm.es (V.R.-D.); joseluis.saldavero@uclm.es (J.L.S.-R)

**Keywords:** micro-electromechanical system (MEMS), resonator, liquid sensing, piezoelectric, aluminium nitride (AlN), grape must fermentation

## Abstract

In this study grape must fermentation is monitored using a self-actuating/self-sensing piezoelectric micro-electromechanical system (MEMS) resonator. The sensor element is excited in an advanced roof tile-shaped vibration mode, which ensures high *Q*-factors in liquids (i.e., *Q* ~100 in isopropanol), precise resonance frequency analysis, and a fast measurement procedure. Two sets of artificial model solutions are prepared, representing an ordinary and a stuck/sluggish wine fermentation process. The precision and reusability of the sensor are shown using repetitive measurements (10 times), resulting in standard deviations of the measured resonance frequencies of ~0.1%, *Q*-factor of ~11%, and an electrical conductance peak height of ~12%, respectively. With the applied evaluation procedure, moderate standard deviations of ~1.1% with respect to density values are achieved. Based on these results, the presented sensor concept is capable to distinguish between ordinary and stuck wine fermentation, where the evolution of the wine density associated with the decrease in sugar and the increase in ethanol concentrations during fermentation processes causes a steady increase in the resonance frequency for an ordinary fermentation. Finally, the first test measurements in real grape must are presented, showing a similar trend in the resonance frequency compared to the results of an artificial solutions, thus proving that the presented sensor concept is a reliable and reusable platform for grape must fermentation monitoring.

## 1. Introduction

The fermentation of grape must into wine involves the interaction between yeasts, bacteria, fungi, and viruses. This complex biochemical process has been recognized and studied since the pioneering investigations of Louis Pasteur in the 1860s. During such a fermentation process, yeasts utilize sugars and other constituents of the grape must for their growth, converting these to alcohol (ethanol), carbon dioxide, and other metabolic end products [1]. A serious problem in winemaking occurs when the yeast growth and the alcoholic fermentation stops prematurely, which results in a wine with residual, unfermented sugar and a concentration of ethanol less than expected. This is indicated in minor deviations of the physical properties, such as density and viscosity, when compared to an ordinary fermentation, and the wine is commonly referred to as being stuck, or sluggish [2,3]. 

There are several approaches to monitor such a fermentation of wine according to different parameters related to the process. El Haloui et al. [4], Nerantzis et al. [5], and Koukolitschek [6] reported on density determination based on differential pressure measurements or flexural oscillators, respectively. By monitoring the CO_2_ release, the fermentation process can also be monitored, as reported in [4]. Another approach is to determine the yeast cell population evolution by means of impedance [7] or turbidity measurements [8]. The propagation velocity in grape must, determined by ultrasound measurements, can also be used for monitoring the fermentation process [9,10]. Lastly, a reflective technique based on fiber optics was reported in [11,12]. Basically, all of these different approaches have their specific drawbacks; some lack enough accuracy, others have only been tested in discrete must samples and, in some cases, the sensor output performance deteriorates dramatically due to an increasing deposition of tartaric acid crystals on the active surface of the sensors [12]. In recent years, cantilever-like micro-electromechanical system (MEMS) resonators have become a reliable platform for various sensing applications [13]. One significant advantage of such resonators is that they can usually be fabricated using silicon micromachining technology and are, therefore, smaller and much cheaper to produce, compared to traditional quartz crystal resonators. So-called “fluidic channel resonators”, in which the fluid passes through a fluidic channel in the moving part of the resonator, is beneficial due to their high-precision capabilities, especially for molecular detection [14], blood coagulation [15], or to quantify other physical properties of fluids, such as mass density or viscosity [16,17]. With such a system only a small amount of liquid (5 μL [18]) is needed and masses can be detected down to the attogram regime [19]. For actuation and sensing, lasers are often used to drive such micro- [20] or nano- [21] mechanical resonators. This approach is considered to be a very accurate and flexible, with the drawback of making the measurement setup bulky, expensive, and mobile integration impracticable. A disadvantage of fluidic channel resonators is their low reusability, which is caused by potential clogging of the fluidic channel. Therefore, such resonators are often designed as one-time use devices, which increases the cost of the overall system. In contrast, solid resonators are often easier to clean, which increases reusability, but generally lacks in precision compared to fluidic channel resonators. The recent improvements in the field of piezoelectric solid MEMS resonators for liquid monitoring purposes predestines such a system for sensing applications, where decent accuracy is required [22,23,24]. The piezoelectric actuation and readout mechanism keeps the sensor device reasonably small and does not require any further laser-based measurement equipment. In this particular field, the question arises whether a micro-machined solid resonator is capable to detect the minor deviations in the physical properties of the grape must during the fermentation process, and whether it offers a promising alternative to the presented measurement approaches. Most recently, Toledo, et al. [25] introduced a phase locked loop-based oscillator circuit in combination with a commercial lock-in amplifier to track the oscillation frequency of the solid resonators [26]. Using this measurement setup, in combination with an evaluation procedure presented in [27], it could be shown that the monitoring of grape must fermentation using solid MEMS resonators is, in principal, possible. 

In this study we will focus on the reliability and the reusability of such a solid resonator and what impact potential contaminations caused have on the sensor characteristics despite the harsh liquid environment. Based on these investigations, an estimation of the precision of the sensor concept is given. Furthermore, different measurement and evaluation procedures are used to validate the data presented in [25]. Finally, test measurements in a real grape must are presented and compared to those of the artificial solutions, thus proving the suitability of the presented concept for grape must fermentation monitoring. 

## 2. Experimental Details

### 2.1. Sensor Specification

**Figure 1:**
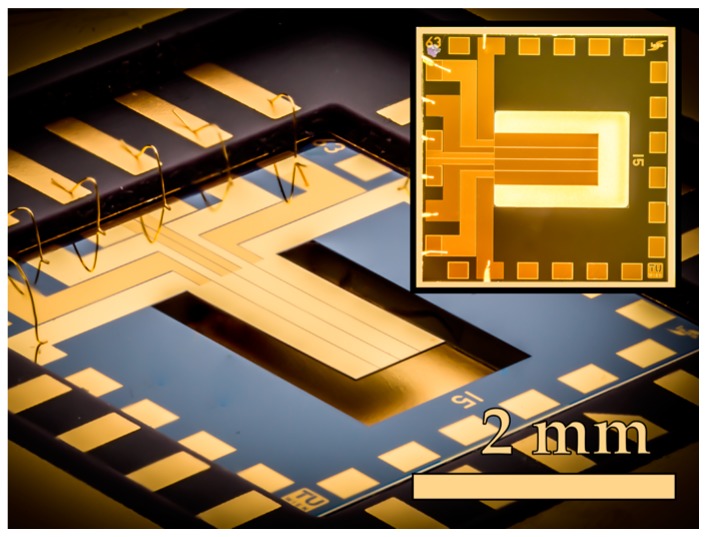
Optical micrograph of the in-house fabricated silicon die (6 mm × 6 mm), containing one piezoelectric actuated plate (dimensions: 2524 μm × 1274 μm × 20 μm) using advanced electrode patterning considering the volume-strain of the modal shape presented in Figure 2.

The piezoelectric MEMS resonator is fabricated on four-inch SOI-wafers and features a length of *L* = 2524 μm, a width of *W* = 1274 μm, and a thickness of *T* = 20 μm, passivated with a SiO_2_/Si_3_N_4_ bi-layer of *t*_iso_ = 250/80 nm. For actuating and sensing, an aluminium nitride (AlN) thin film with a thickness of *t*_AlN_ = 1 μm is sputter-deposited onto the plate surface and is sandwiched between two chromium/gold thin film electrodes with equal thickness for the bottom and top electrode of *t*_be_ = *t*_te_ = 50/450 nm. In Figure 1, a typical sensor chip after packaging and wire bonding with a released single side-clamped resonator, and its top view as an inset, are shown. 

**Figure 2:**
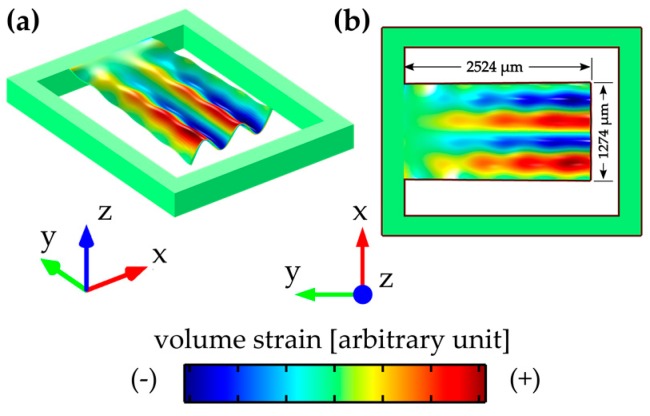
Visualization in side view in (**a**) and in top view in (**b**) for a plate excited in the fourth-order of the roof tile-shaped mode (15-mode). The colored areas on the cantilever surface represent the local volume strain distribution.

**Figure 3:**
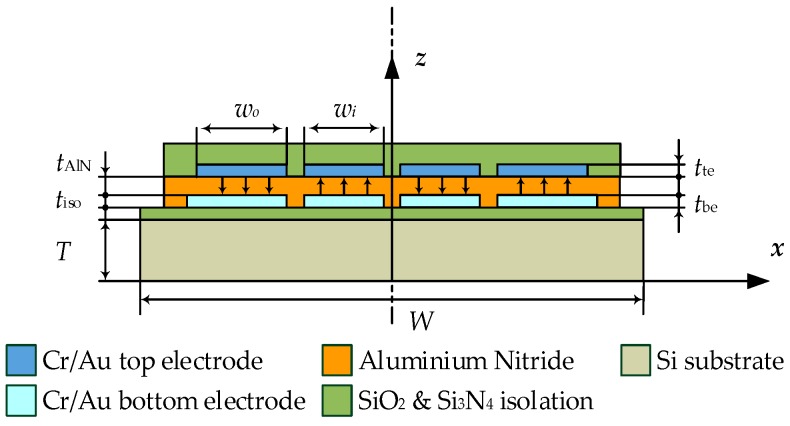
Schematic cross-sectional view on the micro-electromechanical system (MEMS) resonator illustrating the electrode design and the plate support of the 15-mode.

The corresponding mode shape is illustrated by finite-element method (FEM) eigenmode analyses of the fourth-order (15-mode) of the roof tile-shaped mode in Figure 2a in side and, in Figure 2b, in top view. In Figure 3 a schematic cross-sectional view is presented, showing the electrode design optimized for the fourth-order of the roof tile-shaped mode and four electrodes (*w*_o_ = 335 μm, *w*_i_ = 272 μm) with alternating anti-parallel (↓↑↓↑) electric excitation to ensure in addition a collection of almost all generated polarization charges without cancellation. The result of this tailored electrode design is an increased deflection in *z*-direction and, in further consequence, an increased strain related conductance peak as reported in [28], which is one of the major reasons why this oscillation mode is used in this study. Furthermore, it is worth mentioning that the resonance frequencies in liquids do not exceed 600 kHz, which simplifies the electrical measurement procedure and minimized any pressure-induced energy losses by sound waves, which requests compressive fluid properties as reported in [29]. Secondly, high *Q*-factors above 100 are achieved with this type of mode, which facilitates a high sensor sensitivity. To overcome the obstacle of parasitic current caused by the conductivity of the model solutions, the entire sensor element, including bond wires and ceramic package, is passivated with an amorphous silicon dioxide thin film with a thickness of ~4 μm. 

### 2.2. Artificial Model Solutions and Sensing Principle

**Figure 4:**
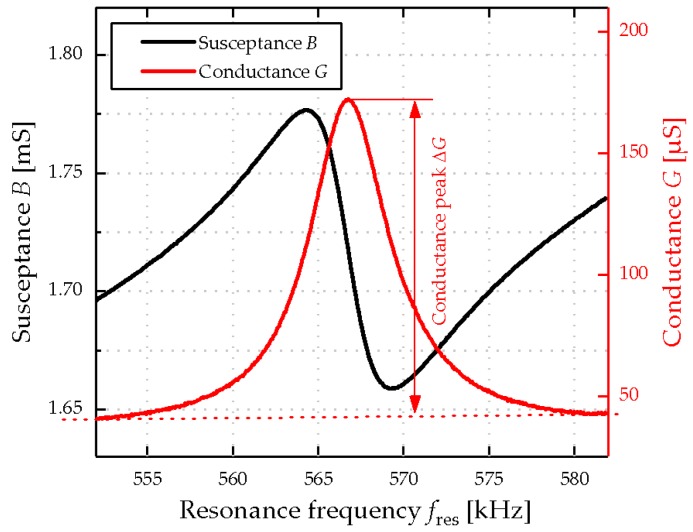
Representative electrical output characteristics given the conductance *G* and susceptance *B* of piezoelectric MEMS resonators when excited in the fourth-order of the roof tile-shaped mode in model solution *N*_1._

Two sets of model solutions are prepared, using a R200D microscale from Sartorius (Goettingen, Germany), representing an ordinary (*N*_1–9_) and a stuck/sluggish (*S*_1–9_) wine fermentation process, consisting of fructose, glucose, glycerol, and ethanol [30]. The specific compositions are listed in Table 2 and Table 3 and show significant differences in the fructose, glucose, and ethanol concentrations, when comparing ordinary (Table 2) and stuck/sluggish (Table 3) wine fermentation processes. The resonator is immersed in these model solutions and actuated in the fourth-order of the roof tile-shaped mode with an Agilent 4294 A impedance analyzer (excitation voltage *V*_exc_ = 500 mV AC, Keysight, Böblingen, Germany). The actuation in the resonance leads to an increased average deflection and to increased strain on the sensor surface, respectively. Due to the piezoelectric effect, polarization charges are generated, which are detected by the impedance analyzer as an increased conductance peak Δ*G*. A typical peak characteristic of the piezoelectric MEMS resonator is shown in Figure 4 when immersed in the model solution *N*_1_. This measurement procedure lasts only ~10 s. Subsequently, the sensor is cleaned with dish soap and isopropanol, followed by a 20 min drying process at room temperature to avoid tartaric acid crystal deposition, as reported in [12].

From these output characteristics, the resonance frequency *f*_res_ and *Q*-factor *Q* are determined using the Butterworth-van Dyke equivalent circuit, in combination with a Levenberg-Marquardt algorithm [31]. Once *f*_res_ and *Q* are obtained for all solutions (*N*_1_–*N*_9_ and *S*_1_–*S*_9_), an evaluation procedure taken from [32] is used to determine the actual density *ρ*_f_ using:

(1) ρf=Im(Zm)−ωresLm+1ωresCm−Re(Zm)+Rmωresd1

Here, *Z*_m_ describes the impedance of the equivalent resonance circuit including a resistance *R*_m_, an inductance *L*_m_ and a capacity *C*_m_ at a certain angular frequency *ω*_res_ in liquid. The constant parameter *d*_1_ is determined in a calibration liquid with known viscosity and density, using:

(2) d1=Im(Zm)−ωresLm_air+1ωresCm_air−d2μfρfωresωresρf,

with:

(3) d2=Re(Zm)−Rm_airμfρfωres

where *R*_m_air_, *L*_m_air_, and *C*_m_air_ are the corresponding unperturbed resonator values in air and are given in Table 1. For this initial calibration process values supplied by the manufacturer for *μ*_f_ and *ρ*_f_ of *N*_4_ and *S*_4_ are used to calculate *d*_1_ and *d*_2_, as shown in Equations (2) and (3), respectively. Once these two constants are obtained, all further values of *ρ*_f_ can be determined using Equation (1). 

### 2.3. Real Grape Must Fermentation

For the investigation of a real fermentation process, *Airen* grapes are processed into grape must with a subsequent filtering process to avoid damaging the sensor by large grape skin particles. After this procedure, the grape must is inoculated with 0.2 g/mL of *Saccharomyces cerevisiae* strain (UCLM S325, Fould-Springer, Ciudad Real, Spain) previously rehydrated, as described in the supplier guidelines. A cylindrical fermenter (mini-Bioreactor Applikon Buitechnology B.V., Delft, The Netherlands) is filled with 3 L of inoculated must, where the temperature was controlled at 28 °C. The fermentation monitoring is carried out by means of the extraction and analysis of 7 mL-samples approximately every 5 h during the following six days. After extraction, the samples were centrifuged for one minute at 1000 rpm (Universal 32R Hettich, Tuttlingen, Germany) and kept refrigerated until analysis.

## 3. Results

**Figure 5:**
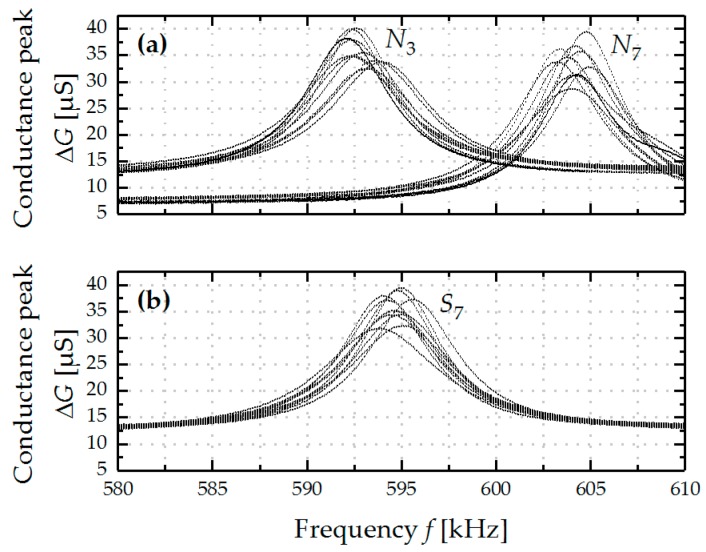
Precision evaluation on the basis of two model solutions of an ordinary fermented grape must in (**a**), and of a stuck fermented grape must in (**b**) at 22 °C.

In Figure 5, the results from the reproducibility measurements are depicted for *N*_3_ and *N*_7_ of an ordinary fermented grape must in Figure 5a, and for *S*_7_ of a stuck fermented grape must in Figure 5b. The measurement procedure, including the cleaning process presented in the previous part of this paper, is repeated for 10 times. Thereby, standard deviations in the resonance frequency *f*_res_ of 0.102% for *N*_3_, 0.085% for *S*_7_ and 0.094% for *N*_7_ are obtained, thus showing a high potential for the targeted application. Higher standard deviations are obtained for the *Q*-factor (~11%) and the conductance peak (12%), which, however, have minor impacts on the final calculation of the density values, indicated by a standard deviation of the calculated density values of 1.2% for *N*_3_, 1.1% for *N*_7_, and 1.0% for *S*_7_. 

**Figure 6:**
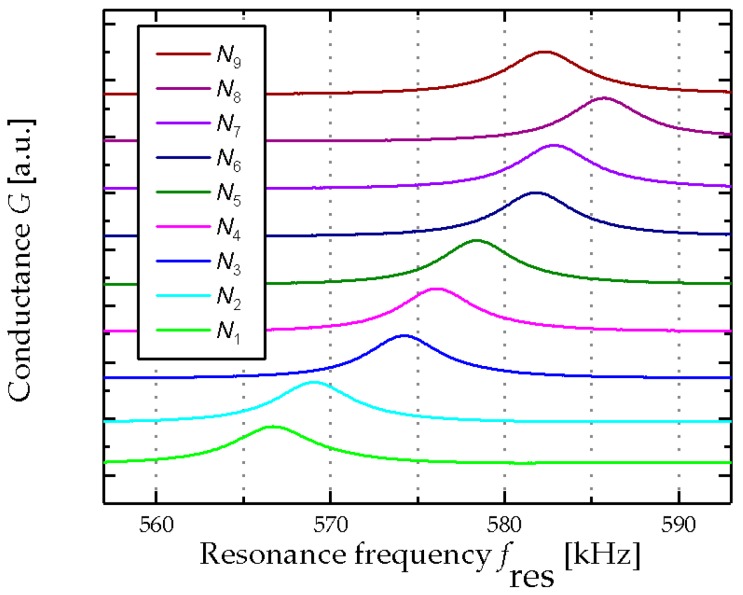
Electrical output characteristics of the piezoelectric MEMS resonator for an ordinary fermentation process *N*_1_–*N*_9_, starting with *N*_1_ at the bottom and all other model solutions (*N*_2_–*N*_9_) stacked above. The *y*-axis is scaled as arbitrary unit [a.u.].

**Figure 7:**
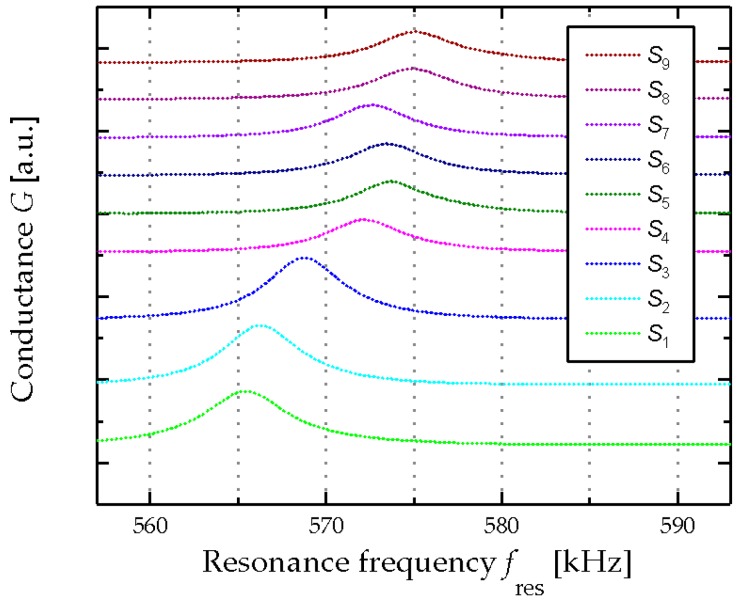
Electrical output characteristics of the piezoelectric MEMS resonator for a stuck fermentation process *S*_1_–*S*_9_, starting with *S*_1_ at the bottom and all other model solutions (*S*_2_–*S*_9_) stacked above. The *y*-axis is scaled as arbitrary unit [a.u.].

The compositions of the artificial wine solutions for an ordinary (*N*_1–9_) wine fermentation process, listed in Table 2, shows a decrease in fructose and glucose concentration from 110 and 100 g/L (*N*_1_) to 2 and 1 g/L (*N*_9_), respectively. Likewise, the glycerol and ethanol concentration are increasing from zero to 9 g/L and 14% *v*/*v*. In the case of a stuck fermented wine process, as it is listed in Table 3, the fructose and glucose concentrations decrease to 50 and 20 g/L, respectively, and remain constant at these values. In parallel, the increase in the ethanol concentration stops as well, and does not exceed a value of 8% (*v*/*v*). These significant changes in the composition of the model solutions affect the resonance response of the MEMS sensor as shown for both an ordinary fermentation in Figure 6 and a stuck fermentation in Figure 7. In the first case, the resonance frequency increases monotonically for the ordinary fermentation (see Figure 6). In contrast, the increase of the resonance frequency during the investigation of the stuck fermentation plateaus at ~575 kHz. 

**Figure 8:**
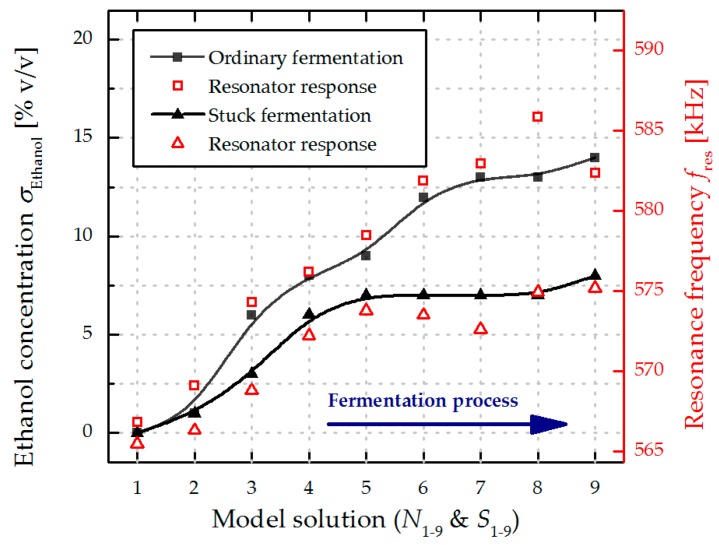
Fifteen-mode frequency response analysis of an ordinary (*N*_1–9_) and a stuck (*S*_1–9_) fermentation process in comparison to the nominal ethanol concentration of the investigated model solutions. The inserted lines serve as guide to the eye.

**Figure 9:**
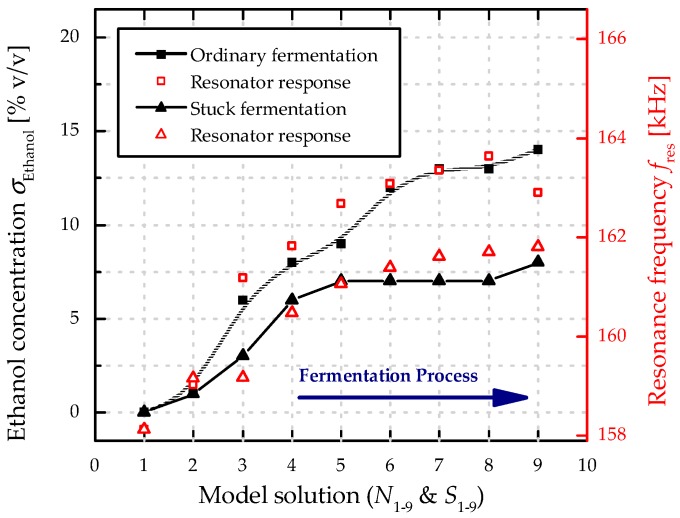
Twelve-mode frequency response analysis of an ordinary (*N*_1–9_) and a stuck (*S*_1–9_) fermentation process in comparison to the nominal ethanol concentration of the investigated model solutions. The inserted lines serve as guide for the eye.

**Figure 10:**
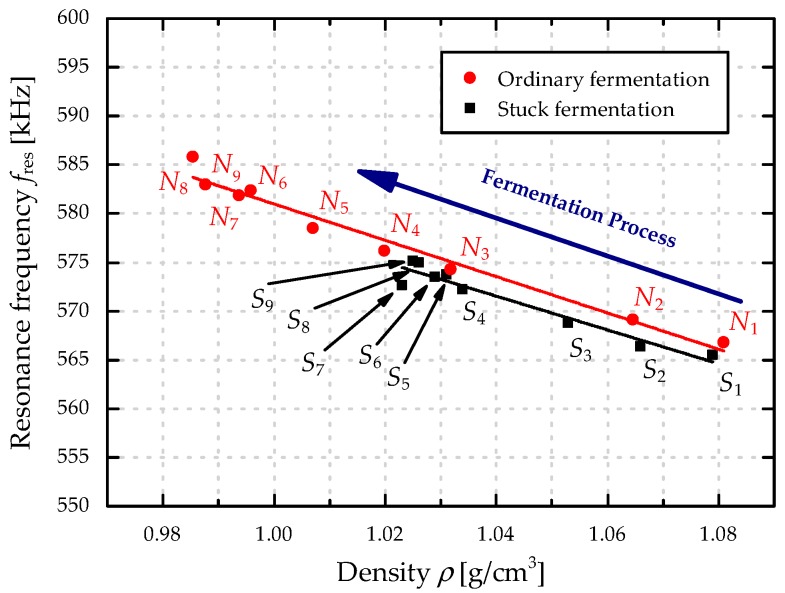
Correlation between the measured resonance frequencies and the corresponding density values for an ordinary (**red**) and a stuck (**black**) fermentation process.

In Figure 8, the results of the resonance response analysis are compared to the ethanol concentration *σ*_Ethanol_ for both sets of model solutions. Significant differences in the resonance frequencies *f*_res_ are evident with increased *σ*_Ethanol_ values when comparing ordinary and stuck fermentation, as well as a good correlation between *σ*_Ethanol_ and *f*_res_. The combination of unfermented sugars and lower concentration of ethanol in a stuck fermentation (see Table 3) leads to a flat resonance characteristics, allowing to detect stuck fermentation. The results from the repeatability measurements of *N*_3_, *N*_7_, and *S*_7_ from Figure 5 are used to estimate the precision of the MEMS sensor. For a further analysis of this particular progress a second resonator with the same geometry is excited in the first roof tile-shaped mode (12-mode). The resonance frequencies in both sets of model solutions amount around 1/4 of the resonance frequency of the 15-mode (~160 kHz) and are depicted in Figure 9, showing a similar characteristics in the frequency response. Compared to the results presented in Figure 7 the absolute change in resonance frequency is also ca. four times lower, leading to a lower sensitivity for the 12-mode. Therefore, all further evaluations are performed using the results from the resonator excited in the 15-mode. In Figure 10 the resonance frequencies for both parameter sets (*N*_1–9_ and *S*_1–9_) as a function of the corresponding density values are depicted, showing a linear increase in *f*_res_ from 567 kHz (*N*_1_) to 582 kHz (*N*_9_), and again a premature stop at ~575 kHz for the stuck fermentation.

**Figure 11:**
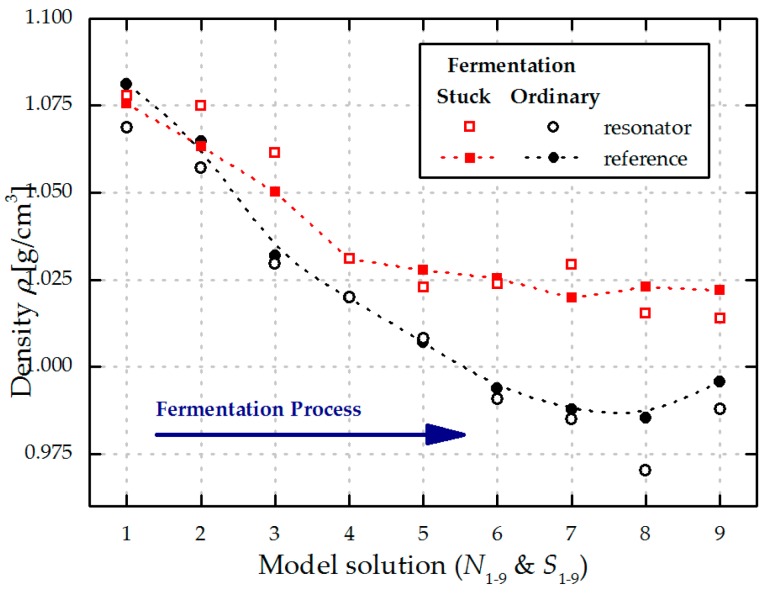
Density values for ordinary (*N*_1–9_) and stuck (*S*_1–9_) fermented model solutions evaluated with the piezoelectric MEMS resonator (unfilled dots) and its reference values obtained with a Stabinger SVM3000 viscometer (filled dots). The inserted dashed lines serve as guides for the eye.

As a next step, the density values of the model solutions are evaluated with a Stabinger SVM3000 viscometer (Anton Paar, Graz, Austria). The results are listed in Table 4 and depicted in Figure 10, showing an almost linear decrease for *N*_1–9_ from 1.081 (*N*_1_) to 0.996 g/cm^3^ (*N*_9_) and a premature stop in the density decrease for *S*_1–9_ with a minimal value of 1.020 g/cm^3^ (*S*_7_). Furthermore, the results of the density determination are compared to those from the Stabinger viscometer in Figure 11, showing low deviations and the possibility to distinguish between ordinary and stuck fermentations at an early stage of the fermentation process.

**Figure 12:**
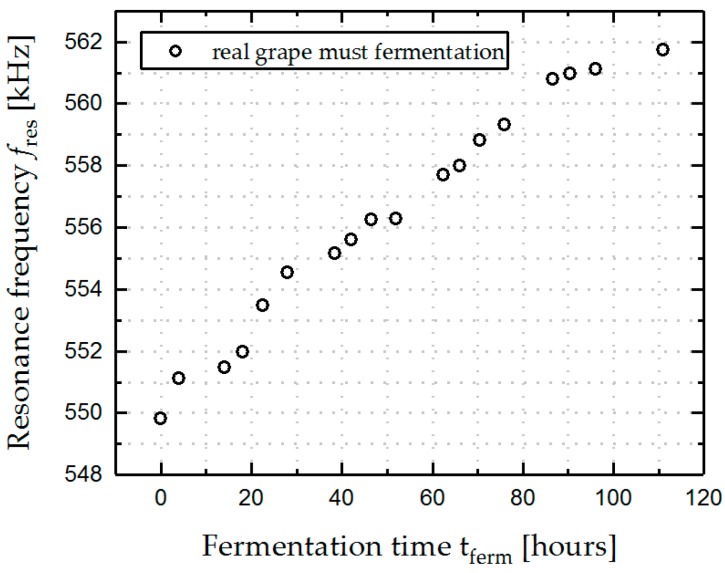
Fifteen-mode frequency response in a real grape over fermentation time at 20 °C.

Finally, the results of the frequency response analysis for real grape must fermentation are presented in Figure 12, showing a similar trend in the change of *f*_res_, compared to those of the artificial solutions presented in Figure 8, which indicates the high potential of the presented sensor system to withstand the harsh conditions during the fermentation process even in real grape must.

## 4. Conclusions

In this paper, an approach to monitor the fermentation processes in winemaking by analyzing the frequency response of a piezoelectric MEMS resonator is presented. The sensor is excited at the fourth-order of the roof tile-shaped mode in several artificial grape must model solutions, representing an ordinary and a stuck wine fermentation process, respectively. During the artificial fermentation, both an increasing ethanol and a decreasing total sugar concentration reduce the density of the grape must despite the counteracting effect of the increasing glycerin concentration. This decrease in density leads to higher resonance frequencies. Due to the high *Q*-factor of the MEMS sensor in liquid media (~100 in isopropanol), even minor shifts in resonance frequency can be detected with high precision, which enables the possibility to monitor these changes of the physical properties during the fermentation processes. Reproducibility and reusability measurement were performed, showing low standard deviations in the resonance frequencies of ~0.1% and moderate deviations in the *Q*-factor (~11%) and conductance peak (~12%). These results show that the resonance frequency is hardly effected by potential surface contaminations. Due to the fast measurement cycle of 10 s and easy cleaning procedure, harsh contaminations, such as the deposition of tartaric acid crystals, could be avoided. Nevertheless, minor surface contaminations reduce the precision of the calculated density values leading to a standard deviation of ~1.1%. Finally, test measurements in real grape must were performed resulting in similar sensor characteristics compared to the results of the model solutions, demonstrating the high potential of the presented sensor concept for grape must fermentation monitoring. 

## Figures and Tables

**Table 1:** Characterization of the resonator in air. *R*_m_air_, *L*_m_air_, and *C*_m_air_ represent the unperturbed resonator in air.

*f*_res_	*Q*	*R*_m_air_	*L*_m_air_	*C*_m_air_
[kHz]	[1]	[kΩ]	[mH]	[fF]
1014.1	329.6	1.365	70.662	348.77

**Table 2:** Composition of the model solutions representing a desired wine fermentation process from *N*_1_ (raw grape must) up to *N*_9_ (ordinary fermented model solution) [30]. The measured values are obtained using a R200D microscale from Sartorius.

Solution	Fructose		Glucose		Glycerol		Ethanol	
-	[g/L]		[g/L]		[g/L]		[% *v*/*v*]	
-	Nom.	Meas.	Nom.	Meas.	Nom.	Meas.	Nom.	Meas.
*N*_1_	110	109.90	100	99.90	0	0	0	-
*N*_2_	90	89.90	80	80.00	0	0	1	-
*N*_3_	70	69.95	30	30.03	5	4.99	6	-
*N*_4_	60	59.97	20	19.99	5	5.00	8	-
*N*_5_	40	39.90	10	9.95	6	5.99	9	-
*N*_6_	20	19.90	2	2.00	7	6.97	12	-
*N*_7_	8	8.02	2	2.00	7	6.99	13	-
*N*_8_	5	5.00	2	2.00	7	6.99	13	-
*N*_9_	2	2.00	1	0.99	9	8.99	14	-

**Table 3:** Composition of the model solutions representing a stuck wine fermentation process from S_1_ (raw grape must) up to S_9_ (stuck fermented model solution) [30]. The measured values are obtained using a R200D microscale from Sartorius.

Solution	Fructose		Glucose		Glycerol		Ethanol	
-	[g/L]		[g/L]		[g/L]		[% *v*/*v*]	
-	Nom.	Meas.	Nom.	Meas.	Nom.	Meas.	Nom.	Meas.
*S*_1_	110	109.90	100	99.98	0	0	0	-
*S*_2_	90	90.00	80	79.98	0	0	1	-
*S*_3_	80	79.98	60	60.01	3	3.00	3	-
*S*_4_	70	69.98	30	29.98	5	4.99	6	-
*S*_5_	66	65.99	27	27.03	5	4.99	7	-
*S*_6_	64	64.00	26	26.06	5	5.01	7	-
*S*_7_	62	61.99	24	24.00	6	5.99	7	-
*S*_8_	58	57.99	22	22.00	6	5.99	7	-
*S*_9_	57	56.99	21	21.00	7	7.01	8	-

**Table 4:** Determined density values (Res.) for ordinary and stuck/sluggish model solutions and their comparison to reference values (Stabinger) obtained with a Stabinger SVM3000 viscometer.

Ordinary				Stuck/Sluggish			
Sol.	Density *ρ* [g/cm^3^]			Sol.	Density *ρ* [g/cm^3^]		
-	Stabinger	Res.	Dev.	-	Stabinger	Res.	Dev.
*N*_1_	1.081	1.082	−0.001	*S*_1_	1.075	1.075	-
*N*_2_	1.065	1.063	0.002	*S*_2_	1.063	1.064	−0.001
*N*_3_	1.032	1.033	−0.001	*S*_3_	1.050	1.050	-
*N*_4_	1.020	1.023	−0.003	*S*_4_	1.031	1.031	-
*N*_5_	1.007	1.010	−0.003	*S*_5_	1.028	1.023	0.005
*N*_6_	0.994	0.994	-	*S*_6_	1.025	1.024	0.001
*N*_7_	0.988	0.989	−0.001	*S*_7_	1.020	1.029	−0.009
*N*_8_	0.985	0.978	0.007	*S*_8_	1.023	1.020	0.003
*N*_9_	0.996	0.995	0.001	*S*_9_	1.021	1.020	0.001

## References

[1] Fleet G.H. (1993). Wine Microbiology and Biotechnology.

[2] Larue F., Lafon-Lafourcade S. (1989). Survival factors in wine fermentation. Alcohol Toxicity in Yeasts and Bacteria.

[3] Munoz E., Ingledew W.M. (1990). Yeast hulls in wine fermentations—A review. J. Wine Res..

[4] El Haloui N., Picque D., Corrieu G. (1988). Alcoholic fermentation in winemaking: On-line measurement of density and carbon dioxide evolution. J. Food Eng..

[5] Nerantzis E., Tataridis P., Sianoudis I., Ziani X., Tegou E. (2007). Winemaking process engineering on line fermentation monitoring—Sensors and equipment. Sci. Technol..

[6] Koukolitschek K. (1993). Verfahren und Vorrichtung zur Präzisen Bestimmung der Alkoholkonzentration in Flüssigkeiten. German Patent.

[7] Pérez M.A., Muñiz R., De La Torre C., García B., Carleos C.E., Crespo R., Cárcel L.M. Impedance spectrometry for monitoring alcoholic fermentation kinetics under wine-making industrial conditions. Proceedings of the XIX IMEKO World Congress Fundamental and Applied Metrology.

[8] Crespo R., Cárcel L., Pérez M., Nevares I., Del Álamo M. Suitable at-line turbidity sensor for wine fermentation supervision. Proceedings of the International Conference on Food Innovation 2010.

[9] Lamberti N., Ardia L., Albanese D., Di Matteo M. (2009). An ultrasound technique for monitoring the alcoholic wine fermentation. Ultrasonics.

[10] Resa P., Elvira L., De Espinosa F.M., González R., Barcenilla J. (2009). On-line ultrasonic velocity monitoring of alcoholic fermentation kinetics. Bioprocess Biosyst. Eng..

[11] Acevedo J.M., Gandoy J.D., Del Río Vázquez A., Freire C.M.-P., Soria M.L. Plastic optical fiber sensor for real time density measurements in wine fermentation. Proceedings of the IEEE Instrumentation and Measurement Technology Conference (IMTC).

[12] Graña C.Q., Acevedo J.M. Experiences in measuring density by fiber optic sensors in the grape juice fermentation process. Proceedings of the XIX IMEKO World Congress Fundamental Applied Metrology.

[13] Boisen A., Dohn S., Keller S.S., Schmid S., Tenje M. (2011). Cantilever-like micromechanical sensors. Rep. Prog. Phys..

[14] Burg T.P., Manalis S.R. (2003). Suspended microchannel resonators for biomolecular detection. Appl. Phys. Lett..

[15] Cakmak O., Ermek E., Kilinc N., Bulut S., Baris I., Kavakli I.H., Yaralioglu G.G., Urey H. (2015). A cartridge based sensor array platform for multiple coagulation measurements from plasma. Lab Chip.

[16] Godin M., Bryan A.K., Burg T.P., Babcock K., Manalis S.R. (2007). Measuring the mass, density, and size of particles and cells using a suspended microchannel resonator. Appl. Phys. Lett..

[17] Khan M.F., Schmid S., Larsen P.E., Davis Z.J., Yan W., Stenby E.H., Boisen A. (2013). Online measurement of mass density and viscosity of pL fluid samples with suspended microchannel resonator. Sens. Actuators B Chem..

[18] Bircher B.A., Duempelmann L., Renggli K., Lang H.P., Gerber C., Bruns N., Braun T. (2013). Real-time viscosity and mass density sensors requiring microliter sample volume based on nanomechanical resonators. Anal. Chem..

[19] Lee J., Shen W., Payer K., Burg T.P., Manalis S.R. (2010). Toward attogram mass measurements in solution with suspended nanochannel resonators. Nano Lett..

[20] Cakmak O., Ermek E., Kilinc N., Yaralioglu G.G., Urey H. (2015). Precision density and viscosity measurement using two cantilevers with different widths. Sens. Actuators A Phys..

[21] Bircher B.A., Krenger R., Braun T. (2016). Automated high-throughput viscosity and density sensor using nanomechanical resonators. Sens. Actuators B Chem..

[22] Kucera M., Wistrela E., Pfusterschmied G., Ruiz-Díez V., Manzaneque T., Sánchez-Rojas J.L., Schalko J., Bittner A., Schmid U. (2014). Characterization of a roof tile-shaped out-of-plane vibrational mode in aluminum-nitride-actuated self-sensing micro-resonators for liquid monitoring purposes. Appl. Phys. Lett..

[23] Kucera M., Wistrela E., Pfusterschmied G., Ruiz-Díez V., Sánchez-Rojas J.L., Schalko J., Bittner A., Schmid U. (2015). Characterisation of multi roof tile-shaped out-of-plane vibrational modes in aluminium-nitride-actuated self-sensing micro-resonators in liquid media. Appl. Phys. Lett..

[24] Pfusterschmied G., Kucera M., Wistrela E., Steindl W., Ruiz-Díez V., Bittner A., Sánchez-Rojas J.L., Schmid U. Piezoelectric response optimization of multi roof tile-shaped modes in MEMS resonators by variation of the support boundary conditions. Proceedings of the 2015 Transducers—2015 18th International Conference on Solid-State Sensors, Actuators and Microsystems (TRANSDUCERS).

[25] Toledo J., Jiménez-Márquez F., Úbeda J., Ruiz-Díez V., Pfusterschmied G., Schmid U., Sánchez-Rojas J.L. (2016). Piezoelectric MEMS resonators for monitoring grape must fermentation. J. Phys. Conf. Ser..

[26] Toledo J., Manzaneque T., Ruiz-Díez V., Jiménez-Márquez F., Kucera M., Pfusterschmied G., Wistrela E., Schmid U., Sánchez-Rojas J.L. Out-of-plane piezoelectric microresonator and oscillator circuit for monitoring engine oil contamination with diesel. Proceedings of the Smart Sensors, Actuators, and MEMS VII, and Cyber Physical Systems.

[27] Toledo J., Manzaneque T., Ruiz-Díez V., Jiménez-Márquez F., Kucera M., Pfusterschmied G., Wistrela E., Schmid U., Sánchez-Rojas J.L. (2016). Comparison of in-plane and out-of-plane piezoelectric microresonators for real-time monitoring of engine oil contamination with diesel. Microsyst. Technol..

[28] Pfusterschmied G., Kucera M., Ruiz-Díez V., Bittner A., Sánchez-Rojas J.L., Schmid U. Multi roof tile-shaped vibration modes in MEMS cantilever sensors for liquid monitoring purposes. Proceedings of the 28th IEEE International Conference on Micro Electro Mechanical Systems (MEMS).

[29] Van Eysden C.A., Sader J.E. (2009). Frequency response of cantilever beams immersed in compressible fluids with applications to the atomic force microscope. J. Appl. Phys..

[30] Jiménez-Márquez F., Vázquez J., Úbeda J., Sánchez-Rojas J.L. (2014). High-resolution low-cost optoelectronic instrument for supervising grape must fermentation. Microsyst. Technol..

[31] Manzaneque T., Hernando J., Rodriguez-Aragon L., Ababneh A., Seidel H., Schmid U., Sánchez-Rojas J.L. (2010). Analysis of the quality factor of AlN-actuated micro-resonators in air and liquid. Microsyst. Technol..

[32] Pfusterschmied G., Kucera M., Wistrela E., Manzaneque T., Ruiz-Díez V., Sánchez-Rojas J.L., Bittner A., Schmid U. (2015). Temperature dependent performance of piezoelectric MEMS resonators for viscosity and density determination of liquids. J. Micromech. Microeng..

